# Sex disparities of the effect of the COVID-19 pandemic on mortality among patients living with tuberculosis in the United States

**DOI:** 10.3389/fpubh.2024.1413604

**Published:** 2024-06-18

**Authors:** Huan Deng, Yishan Liu, Fan Lv, Xiaofeng Li, Mingyan Qi, Yajing Bo, Sikai Qiu, Xinyuan He, Fanpu Ji, Qing-Lei Zeng, Ning Gao

**Affiliations:** ^1^National and Local Joint Engineering Research Center of Biodiagnosis and Biotherapy, The Second Affiliated Hospital of Xi'an Jiaotong University, Xi'an, China; ^2^Department of Infectious Diseases, The Second Affiliated Hospital of Xi'an Jiaotong University, Xi'an, China; ^3^School of Mathematics and Statistics, Xi'an Jiaotong University, Xi'an, China; ^4^Center for Infectious Diseases, The Second Affiliated Hospital of Air Force Medical University, Xi'an, China; ^5^Department of Clinical Medicine, The Second Affiliated Hospital of Xi'an Jiaotong University, Xi'an, China; ^6^Provincial Research Center, Shaanxi Provincial Clinical Medical Research Center of Infectious Diseases, Xi'an, China; ^7^Global Health Institute, School of Public Health, Xi'an Jiaotong University Health Science Center, Xi'an, China; ^8^Key Laboratory of Surgical Critical Care and Life Support (Xi'an Jiaotong University), Ministry of Education, Xi'an, China; ^9^Department of Infectious Diseases, The First Affiliated Hospital of Zhengzhou University, Zhengzhou, Henan, China

**Keywords:** tuberculosis, COVID-19, age-standardized mortality rate, excess mortality, female

## Abstract

**Background:**

We aimed to determine the trend of TB-related deaths during the COVID-19 pandemic.

**Methods:**

TB-related mortality data of decedents aged ≥25 years from 2006 to 2021 were analyzed. Excess deaths were estimated by determining the difference between observed and projected mortality rates during the pandemic.

**Results:**

A total of 18,628 TB-related deaths were documented from 2006 to 2021. TB-related age-standardized mortality rates (ASMRs) were 0.51 in 2020 and 0.52 in 2021, corresponding to an excess mortality of 10.22 and 9.19%, respectively. Female patients with TB demonstrated a higher relative increase in mortality (26.33 vs. 2.17% in 2020; 21.48 vs. 3.23% in 2021) when compared to male. Female aged 45–64 years old showed a surge in mortality, with an annual percent change (APC) of −2.2% pre-pandemic to 22.8% (95% CI: −1.7 to 68.7%) during the pandemic, corresponding to excess mortalities of 62.165 and 99.16% in 2020 and 2021, respectively; these excess mortality rates were higher than those observed in the overall female population ages 45–64 years in 2020 (17.53%) and 2021 (33.79%).

**Conclusion:**

The steady decline in TB-related mortality in the United States has been reversed by COVID-19. Female with TB were disproportionately affected by the pandemic.

## 1 Introduction

Tuberculosis (TB) is one of the leading causes of disease burden and death worldwide. The global cost of TB control is up to US $1 billion annually (1). In 2014, the World Health Organization (WHO) proposed a strategy to combat TB, which strives to achieve a 95% reduction in TB mortality from 2015 to 2035, and to reduce individual and systemic healthcare costs related to TB (2). Between 2015 and 2018, the number of global TB deaths fell by 11%, which fell short of the projected decrease represented by this WHO goal (3). In 2019, there were an estimated 10 million new TB cases worldwide. As a low-burden TB country, the United States has a stabile TB incidence at ~30 new cases per 1 million people after years of slow incidence decline (4). However, this incidence still does not reach the goal of a 20% drop in incidence between 2015 and 2020 set forth by the WHO TB strategy (2).

Unfortunately, the COVID-19 pandemic has had a devastating impact on TB control, and in many aspects reversed the global progress made in the fight against TB. Many areas of the world have seen declines in TB testing and reporting of cases, and some have even observed the first increase in TB-related mortality in the past decade (5–7). In countries with a high burden of TB, the disruption of healthcare services due to reduced outbreaks, redistribution of human and material resources, and reduced social support could lead to a projected 20% increase in the number of TB-related deaths in the next 5 years (6, 8–10). Despite efforts by some TB centers to keep healthcare services fully operational during the pandemic, it has proven impossible to avoid a large decrease in the number of diagnosed cases and an increased number of patients lost to follow-up or dying during this time (11).

The substantial impact of the COVID-19 pandemic on TB control is highlighted by a sharp decline in case reporting and the squeeze on medical resources, which ultimately lead to a decline in the ability to provide timely diagnosis and treatment resulting in poor outcomes and increased TB-related mortality (12–14). On the other hand, co-infection of tuberculosis and COVID-19 was associated with an increased risk of unfavorable clinical outcomes, as reported by Aiello et al., which may have been related to immune responses (15). Particularly, the decreased immune response in older adults may have limited the ability to contain pathogen growth (16). As of yet, there has been little evidence published on the impact of the COVID-19 pandemic on TB in the United States, particularly with respect to possible gender differences. Therefore, we aimed to assess the impact of the COVID-19 pandemic on TB mortality in the United States with focus on sex differences using nationally representative data.

## 2 Methods

### 2.1 Data sources

The current study represented a population-based time series analysis combined with predictive analysis performed on data from decedents in the United States. Data were analyzed from decedents who had died from January 1, 2006 to December 31, 2021. Data were obtained from the National Vital Statistics System (NVSS) dataset through the Center for Disease Control and Prevention Wide-Ranging Online Data for Epidemiologic Research (CDC-WONDER) website. This database contained annual death data of more than 99% of decedents in 50 U.S. states and the District of Columbia. The most recent data available were used (updated most recently on April 16, 2022). We also collected demographic data including age, sex, and race/ethnicity. Since all data from NVSS were publicly available and completely deidentified, the study did not seek approval from the Institutional Review Board. The study is compliant with the Strengthening the Reporting of Observational Studies in Epidemiology (STROBE) guidelines.

### 2.2 Definition

The targeted population was decedents aged 25 years or older with TB as one of the causes of death. The study period was defined between January 1, 2006 and December 31, 2021. We identified TB and COVID-19 cases using criteria detailed in the 10th Edition of the International Classification of Diseases (ICD-10). The stratified analyses were classified by age (25–44, 45–64, and ≥65 years) and sex (male and female). We also stratified the data by racial/ethnic groups defined by the U.S. Office of Management and Budget, including non-Hispanic Alaska Indians/American Natives, non-Hispanic Asians, non-Hispanic Blacks, Hispanics, and non-Hispanic Whites. In 2021, the racial/ethnic component on the CDC Miracle website changed when the new subgroup “multiple races” was introduced. Thus, the subgroup analysis by race/ethnicity included only data through 2020.

### 2.3 Statistical analysis

We used age-standardized mortality rates (ASMR), using the 2010 U.S. Census as a reference, to ensure comparability across study years. Crude mortality rate (number of TB-related deaths/total population) was calculated and age-standardized using direct methods. In the direct approach, we stratified the number of TB-related deaths and the reference population by 10 years (from 25 to 64 years old, then ≥65 years old).

Using mortality data available from 2006 and 2019, TB-related mortality and general population mortality were predicted to determine the expected ASMR between 2020 and 2021. Autoregressive moving average model (ARMA), autoregressive integrated moving average model (ARIMA), polynomial regression, and linear regression were tested. Given the trend in mortality from 2006 to 2019, we chose polynomial regression with least squares for fitting. Model fitness was assessed using R square (Supplementary Table 1). The impact of the pandemic was determined by calculating the percentage difference between the expected and observed mortality rates, such that the excess mortality rate was defined as:


$$\begin{array}{l} \left(observed-predicted\;value\right)/predicted\;value\;x\;100\% \end{array}$$

A time-trend analysis was performed using a connected-point analysis via piecewise regression with Monte Carlo permutation tests to estimate the direction, magnitude, and significance of trends, and to determine the segmentation of trends. All analyses were performed using Joint Point Trend Analysis software (version 4.9.1.0; National Cancer Institute, Bethesda, MD), PyCharm3.9.0 (prediction analysis), and R 4.0.2 software (all other analyses and data cleaning). The significance threshold was defined as a two-tailed *p*-value of < 0.05.

## 3 Results

### 3.1 Population and characteristics of analyzed deaths

A total of 18,628 TB-related deaths among adults aged 25 years or older were recorded between 2006 and 2021 (Table 1). The older adults group (≥65 years) represented the greatest percentage of these deaths at 65.61%, followed by 28.21% in the 45–64 years group, with the lowest percentage occurring in the 25–44 years age group (6.18%). With respect to sex, the mortality of males was greater than that of females (63.18 vs. 36.82%, respectively). Finally, with respect to race/ethnicity, nearly half of the analyzed TB-related deaths occurred in non-Hispanic Whites (8,008; 46.6%), followed by non-Hispanic Blacks (3,153; 18.4%), non-Hispanic Asians (2,882; 16.8%), and Hispanics (2,709; 15.8%). American Indian/Alaska Natives represented only 2.45% of the deaths (Table 1).

**Table 1:** Characteristics of TB-related death in the United States, 2006–2021.

	**Deaths (%)**	**Deaths (%)**	**Deaths (%)**	**Deaths (%)**	**Deaths (%)**
	**2006–2021**	**2006**	**2019**	**2020**	**2021**
**Overall**	18,628 (100.0)	1,359 (100.0)	1,106 (100.0)	1,275 (100.0)	1,456 (100.0)
**Age**					
25–44	1,151 (6.18)	87 (6.40)	72 (6.51)	85 (6.66)	109 (7.49)
45–64	5,256 (28.21)	372 (27.37)	279 (25.22)	359 (28.16)	409 (28.09)
≥65	12,221 (65.61)	900 (66.23)	755 (68.26)	831 (65.18)	938 (64.42)
**Sex**					
Female	6,858 (36.82)	512 (37.67)	391 (35.35)	480 (37.65)	523 (35.92)
Male	11,770 (63.18)	847 (62.33)	715 (64.65)	795 (62.35)	933 (64.08)
**Race/ethnicity** ^§^					
Non-Hispanic AI/AN	420 (2.45%)	35 (2.58)	19 (1.72)	32 (2.51)	NA
Non-Hispanic Asians	2,882 (16.8%)	175 (12.88)	247 (22.33)	253 (19.84)	NA
Non-Hispanic Blacks	3,153 (18.4%)	298 (21.93)	176 (15.91)	215 (16.86)	NA
Hispanics	2,709 (15.8%)	205 (15.08)	162 (14.65)	229 (17.96)	NA
Non-Hispanic Whites	8,008 (46.6%)	646 (47.53)	502 (45.39)	546 (42.82)	NA

AI/AN, American Indian/Alaska Native.

^§^n = 17,172 between 2006–2020.

### 3.2 The impact of the COVID-19 pandemic on mortality in patients with TB and in the general population

**Figure 1:**
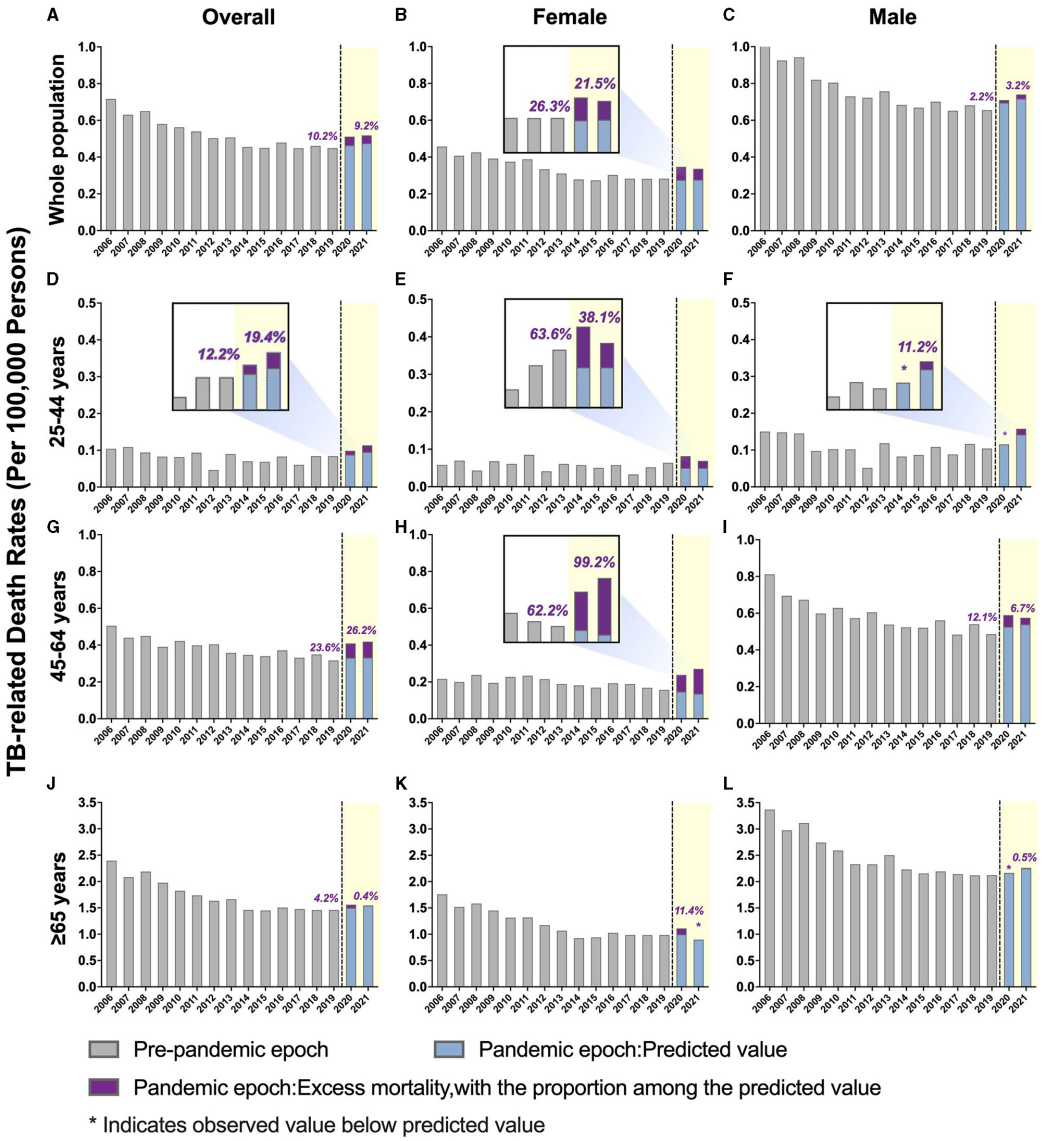
Temporal trends of all-cause mortality in people with tuberculosis (TB) and excess mortality during the COVID-19 pandemic by sex, further stratified by age. **(A–C)** Overall TB-related mortality: **(A)** males and females; **(B)** females only; **(C)** males only. **(D–F)** TB-related mortality, ages 25–44 years: **(D)** male and female; **(E)** female only; **(F)** male only. **(G–I)** TB-related mortality, ages 45–64 years: **(G)** male and female; **(H)** female only; **(I)** male only. **(J–L)** TB-related mortality, ages ≥ 65 years: **(J)** males and females; **(K)** females only; **(L)** males only. The blue bars represent the predicted mortality rate. Purple bars represent the excess mortality associated with COVID-19.

The TB-related ASMR was 0.72 per 100,000 persons in 2006, which decreased to 0.45 per 100,000 persons by 2019 (Figure 1 and Table 2). A rise in mortality rate was observed during the pandemic, with an ASMR of 0.51 per 100,000 persons in 2020 and 0.52 per 100,000 persons in 2021, corresponding to excess mortality of 10.22 and 9.20%, respectively. The overall ASMRs of adults in the U.S. general population stratified by age and sex are shown in Table 3 and Supplementary Figure 1.

**Table 2:** Age-standardized mortality rates, U.S. adults with TB, 2006–2021.

	**Age-standardized mortality rate (per 100,000 persons)**							
	**Pre-pandemic referent epoch 2006**	**Pre-pandemic referent epoch 2019**	**Pandemic epoch 1 2020**			**Pandemic epoch 2 2021**		
	**Observed**	**Observed**	**Observed**	**Predicted (95% CI)**	**% difference** ^*^	**Observed**	**Predicted (95% CI)**	**% difference** ^*^
**Overall**								
Multiple cause of death^∫^	0.72	0.45	0.51	0.46 (0.43–0.50)	10.22	0.52	0.48 (0.43–0.52)	9.20
Multiple cause of death^∫∫^ (non-COVID-19 related)	0.72	0.45	0.46	0.46 (0.43–0.50)	−0.19	0.45	0.48 (0.43–0.52)	−4.6
Underlying cause of death^∫∫∫^	0.36	0.23	0.25	0.23 (0.21–0.26)	8.35	0.24	0.24 (0.21–0.27)	2.44
**Age**								
25–44 years	0.10	0.08	0.10	0.09 (0.06–0.12)	12.16	0.113	0.10 (0.06–0.13)	19.43
45–64 years	0.51	0.32	0.41	0.33 (0.29–0.37)	23.62	0.42	0.332 (0.27–0.39)	26.23
≥65 years	2.40	1.46	1.56	1.50 (1.36–1.63)	4.23	1.55	1.54 (1.37–1.72)	0.44
**Sex**								
Female	0.46	0.28	0.35	0.28 (0.24–0.32)	26.33	0.34	0.28 (0.23–0.33)	21.48
Male	1.05	0.66	0.71	0.70 (0.63–0.76)	2.17	0.74	0.72 (0.64–0.80)	3.23
**Female by age**								
25–44 years	0.06	0.06	0.08	0.05 (0.03–0.07)	63.59	0.07	0.05 (0.03–0.07)	38.07
45–64 years	0.22	0.16	0.24	0.15 (0.12–0.18)	62.17	0.26	0.14 (0.09–0.18)	99.16
≥65 years	1.76	0.98	1.11	1.00 (0.86–1.14)	11.42	1.02	1.03 (0.85–1.21)	−1.37
**Male by age**								
25–44 years	0.15	0.10	0.12	0.13 (0.09–0.17)	−9.87	0.16	0.14 (0.09–0.19)	11.21
45–64 years	0.81	0.49	0.59	0.53 (0.46–0.59)	12.10	0.58	0.54 (0.45–0.63)	6.71
≥65 years	3.37	2.12	2.17	2.193 (1.97–2.42)	−1.20	2.26	2.25 (1.96–2.54)	0.52

^*^Denotes % difference between predicted and observed values.

^∫^Multiple cause of death: Deaths caused by various causes, including TB. ^∫∫^Excluded decedents with COVID-19 infection as the multiple cause of death. ^∫∫∫^Underlying cause of death: The disease or injury which initiated the series of events leading directly to death (i.e., decedents with TB listed as the primary cause of death).

**Table 3:** Age-standardized mortality rates, U.S. general adult population, 2006–2021.

	**Age-standardized mortality rate (per 100,000 persons)**							
	**Pre-pandemic referent epoch 2006**	**Pre-pandemic referent epoch 2019**	**Pandemic epoch 1 2020**			**Pandemic epoch 2 2021**		
	**Observed**	**Observed**	**Observed**	**Predicted (95% CI)**	**% difference** ^*^	**Observed**	**Predicted (95% CI)**	**% difference** ^*^
**Overall**								
	1,248.52	1,137.37	1,329.16	1,157.80 (1,136.59–1,179.0)	14.80	1,353.23	1,163.86 (1,136.31–1,191.41)	16.27
**Age**								
25–44 years	151.12	165.65	205.36	171.814 (159.51–184.12)	19.52	237.02	174.113 (154.323–193.903)	36.13
45–64 years	640.62	610.86	723.24	606.48 (597.40–615.57)	19.25	797.73	597.57 (582.96–612.17)	33.50
≥65 years	4,717.08	4,184.90	4,847.56	4,158.94 (4,040.41–4,277.46)	16.56	4,754.12	4,102.48 (3,911.90–4,293.06)	15.88
**Sex**								
Female	1,062.35	961.62	1,110.52	953.97 (929.52–978.42)	16.41	1,121.90	938.03 (898.72–977.34)	19.60
Male	1,489.54	1,345.57	1,586.16	1,344.28 (1,316.38–1,372.17)	17.99	1,624.35	1,332.38 (1,287.53–1,377.23)	21.91
**Female by age**								
25–44 years	103.72	111.25	134.11	114.06 (107.21–120.92)	17.57	156.33	114.45 (103.42–125.48)	36.59
45–64 years	486.69	461.92	534.73	454.96 (445.94–463.98)	17.53	593.83	443.85 (429.35–458.36)	33.79
≥65 years	4,182.61	3,707.35	4,267.33	3,676.96 (3,557.68–3,796.24)	16.06	4,160.04	3,617.93 (3,426.15–3,809.71)	14.98
**Male by age**								
25–44 years	198.63	219.34	275.61	228.71 (210.70–246.73)	20.51	316.57	232.77 (203.81–261.73)	36.00
45–64 years	803.91	768.22	922.09	766.21 (756.09–776.32)	20.35	1,012.39	759.12 (742.86–775.38)	33.36
≥65 years	5,510.09	4,811.10	5,603.12	4,789.33 (4,670.63–4,908.43)	16.99	5,530.23	4,735.34 (4,533.16–4,926.52)	16.79

^*^Denotes % difference between predicted and observed values.

### 3.3 Sex disparities in the impact of the COVID-19 pandemic on TB-related deaths

A downward trend in TB-related mortality rate in males was evident prior to the pandemic, dropping from 1.05 per 100,000 persons in 2006 to 0.66 per 100,000 persons in 2019. This rate increased during the pandemic, with an annual percent change (APC) of 6.7% (predicted 95% CI: −6.5 to 21.7%; *p* > 0.05). The ASMRs were 0.710 per 100,000 persons in 2020 and 0.740 per 100,000 persons in 2021, with excess mortality of 2.17 and 3.23%, respectively (Figure 1 and Supplementary Table 2).

In contrast, the TB-related mortality rates in females increased during the pandemic, with ASMRs of 0.35 per 100,000 persons in 2020 and 0.336 per 100,000 persons in 2021 (Table 2 and Supplementary Figure 2). Notably, the percent difference between the observed and predicted mortality in female patients with TB during the pandemic was higher than in the general female population in 2020 (26.325 vs. 16.41%, respectively) and 2021 (21.48 vs. 19.60%, respectively; Tables 2, 3).

### 3.4 By age group

The pre-pandemic TB-related ASMR of the 45–64 years age group showed a significant downward trend, decreasing from 0.51 per 100,000 persons in 2006 to 0.32 per 100,000 persons in 2019 (APC: −2.8%; *p* < 0.05). However, during the pandemic, there was an upward trend in mortality with an APC of 16.8% (95% CI: −2.2 to 39.5%; *p* > 0.05). The observed mortality rates were 0.41 per 100,000 persons in 2020 and 0.42 per 100,000 persons in 2021, corresponding to excess mortality of 23.62 and 26.23%, respectively (Figure 1, Table 2, and Supplementary Table 2). Furthermore, the excess mortality observed in this group was higher in 2020 compared to the general population (23.62 vs. 19.25%, respectively; Tables 2, 3). Mortality rates within the 25–44 and ≥ 65 years age groups also decreased before the pandemic, but the trend was not significant (Figure 1 and Table 2).

### 3.5 By sex-age subgroup

**Figure 2:**
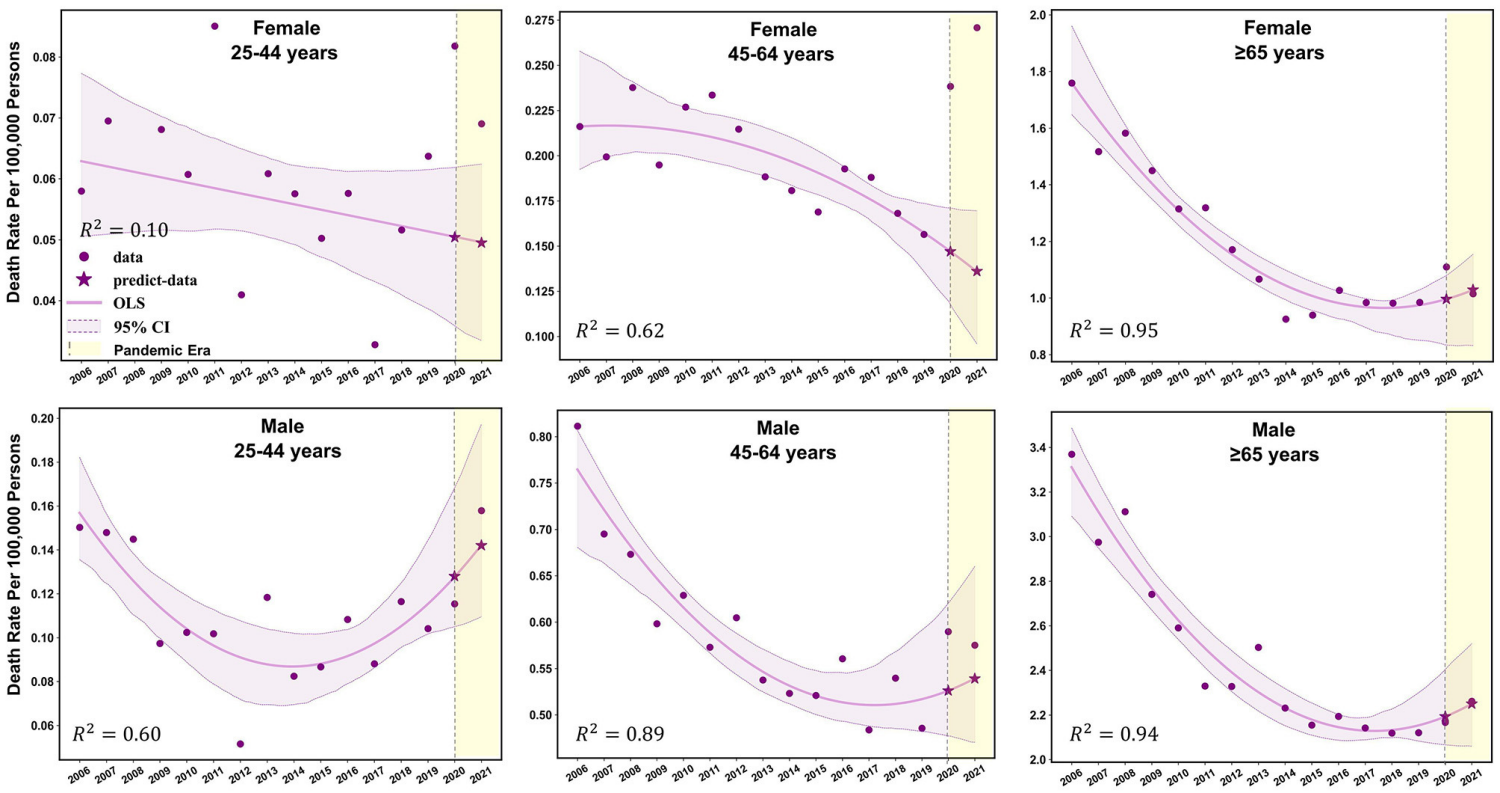
Observed and predicted age-standardized mortality rates for TB-related deaths before and during the COVID-19 pandemic among different sex-age subgroups.

Females aged 45–64 years showed a significant decrease in mortality pre-pandemic (APC −2.2%; *p* < 0.05), and mortality surged with an APC of 22.8% (95% CI: −1.7 to 68.7%; *p* > 0.05) during the pandemic (Supplementary Table 3). The observed mortality rates of female patients with TB in this age group were 0.24 per 100,000 persons and 0.26 per 100,000 persons in 2020 and 2021, respectively (Figures 1, 2, and Table 2). These data represent excess mortalities of 62.17 and 99.16% in 2020 and 2021, respectively, Which were higher than the excess mortalities in the general female population aged 45–64 years in 2020 (17.53%) and 2021 (33.79%; Tables 2, 3). Female patients with TB and aged 25–44 years also showed high excess mortalities in 2020 (63.59%) and 2021 (38.07%), both higher than that in general female population of the same age group (Figures 1, 2, Tables 2, 3). The increase in male mortality was modest throughout the pandemic, and the mortality rate observed across all ages for males was within the 95% CI of the predicted mortality rate (Figure 2).

### 3.6 By race/ethnicity group

Overall mortality rates decreased across all racial/ethnic groups prior to the pandemic and demonstrated a mild increase during the pandemic (Supplementary Figure 3). In 2020, the observed mortality rate increased significantly in the non-Hispanic white subgroups to higher than the predicted mortality rate, representing an excess mortality of 10.29%. Observed mortality rates for racial/ethnic subgroups of non-Hispanic black, Hispanic, non-Hispanic Asian, and non-Hispanic American Indian/Alaska Native were within the 95% CI of predicted mortality rates (Supplementary Table 4 and Supplementary Figure 3).

## 4 Discussion

This study analyzed 18,628 TB-related deaths occurring between 2006 and 2021 in the United States, and in doing so demonstrated the significant impact of the COVID-19 pandemic with respect to the progress made in eliminating TB in this country. During the pandemic, TB-related mortality rates significantly increased for the first time since 2006, and the observed TB-related mortality rates were significantly higher than the projected mortality rates for all age and sex subgroups in 2020 and 2021. Our data support the theory that since the early 2020's, the disruption of TB management has shifted healthcare priorities to COVID-19, which has led to the reversal of the decline of TB-related mortality. When further stratified by age and sex, there was a profound and steep increase in TB-related mortality among females aged 45–64 years, and a more moderate increase of the same in females aged 25–44 years, both of which exceeded mortality in the same female age groups of the general population. This phenomenon was observed despite the overall excess mortality being lower during the pandemic (10.22 vs. 14.800% in 2020 and 9.194 vs. 16.271% in 2021). Taken together, the precipitous increase in mortality rates suffered by young and middle-aged females with TB highlights the need for additional resources to address the underlying social and economic burden experienced by female patients and to mitigate the effects that will undoubtedly linger for years following the pandemic.

The challenges associated with TB control have been recognized from the start of the pandemic. Recent studies have shown that TB patients utilized healthcare services less during the pandemic than in previous years. The implementation of emergency measures such as home quarantine, cancellation of public transport, closure of public places, TB hospitals being designated as COVID-19 hospitals, and allocation of medical staff to COVID-19 wards has led to the interruption of health services, resulting in a significant decrease in TB diagnosis and reporting and an increase in TB-associated mortality (17–22). We quantified excess mortality in patients with TB during the pandemic and found a higher number of excess deaths in the female TB patient population, suggesting that more cases and opportunities for disease management were being missed in female TB patients. Soko et al. also found that females were most affected by TB service disruption and had the most pronounced decline in TB case notifications (20). Although service disruptions led to decreased disease detection, the proportion of females in the TB patient population increased more than before the pandemic (23). In addition to the impact of healthcare disruption, fear and stigma surrounding COVID-19, lack of access to adequate healthcare, and social determinants like gender inequality also likely contributed to the decrease in TB diagnosis and the increase in mortality among female TB patients.

We also observed an upward trend in deaths in male TB patients, but this was not significant as the observed mortality rate was within the 95% CI of the model predicted value. There are data suggesting that male gender is an independent risk factor for death related to TB and COVID-19 coinfection (24). In addition to male sex, the increased mortality was also associated with the presence of TB sequelae (25). These data suggest that the risk of death after COVID-19 infection is increased for both old and new TB cases. The risk of death was 1.51-fold in TB patients with COVID-19 diagnosed before the pandemic and 2.7-fold in newly-diagnosed TB patients with COVID-19 (26). Certainly, there is also evidence that the impact of COVID-19 coinfection on the TB clinical course seems modest, except for in cases of death where no serious clinical deterioration was observed (27). This seems to be explained by the fact that the effects of COVID-19 on TB disease course can be controlled through appropriate clinical care. In fact, COVID-19 mortality and incidence rates are low in the U.S. as well as in many Asian countries with high TB burden, which may be associated with immunity produced by long-term TB infection (28). Ahmed et al. reported that established pulmonary TB may have a protective effect against COVID-19, which may also support this speculation (29). However, TB has also been suggested as a risk factor for disease progression in COVID-19 (30), as evidenced by the higher mortality, shorter time to death, and longer recovery time after acquisition of previous TB infection compared with patients without TB (31). The reports by Petrone et al. and Najafi et al. also indicated that the inability of the immune system to *in vitro* respond to SARS-CoV-2 and *M. tuberculosis* antigens in TB-COVID-19 co-infected subjects (32, 33).

Furthermore, vaccination as a key social prevention strategy for TB may also have implications for COVID-19. A Japanese study found that routine infant BCG coverage was protective against the transmission of COVID-19 (34). This may be related to the effect of BCG vaccination on COVID-19-related immunity (35), but there has been controversy whether BCG vaccination plays any role. Lerm et al. suggest that COVID-19 does not cause serious disease in most unvaccinated young people and healthy people, and that high-quality studies on the effects of BCG vaccination on COVID-19 incidence, disease course, and transmission are needed to determine whether and how BCG could help control the outbreak (36).

In any case, the disruption of TB services due to medical and social measures put in place for management of COVID-19 is one of the main reasons for the sharp increase in TB-related deaths, with females being most affected. The U.S. government is a very important force in the global fight against TB, and it also provides the largest funds for global tuberculosis control in the world. Currently, TB is a greater long-term threat than COVID-19, and the government must strengthen services and support for TB patients, especially for disproportionately affected groups.

The main strength of our study is the use of a nationwide database to quantify the impact of the COVID-19 pandemic on the TB mortality rate in the United States and associated sex differences. The database we used had comprehensive records of deaths such that our selection bias was low. Secondly, a suitable model was utilized to predict general mortality rates during the pandemic, which provided the basis for further identification of excess mortality. In addition, our subgroup analysis highlighted the differences in excess mortality between sexes, ages, and ethnic groups. Our main limitation is that the death data are susceptible to biases such as underreporting, which could lead to underestimation of the excess mortality rate. For all analyses, data associated to clinical characteristics, cs, including occupation, education, and income, were not available. Therefore, additional studies are needed to investigate correlates and potential determinants of the trends in excess mortality in female patients aged 45–64 year found in our analyses of vital statistics. Furthermore, no data on race/ethnicity were used from 2021 because a new category was added (more than one race) during this year.

## 5 Conclusion

The steady decline in TB-related mortality in the United States has unfortunately been reversed by COVID-19. Female patients with TB were disproportionately affected during the pandemic, likely owing to care gaps and health disparities experienced by females that were accentuated during the pandemic. TB is a more substantial long-term threat to global health than COVID-19; as such, effective public health infrastructure and concrete policy changes are instrumental to ensuring an equitable and resilient health care delivery system to help prevent the deaths of vulnerable populations with TB.

## Data availability statement

The original contributions presented in the study are included in the article/Supplementary material, further inquiries can be directed to the corresponding authors.

## Ethics statement

Written informed consent was not obtained from the individual(s) for the publication of any potentially identifiable images or data included in this article because Since all data from NVSS were publicly available and completely de-identified, the study did not seek approval from the institutional review board.

## Author contributions

HD: Methodology, Writing – review & editing, Writing – original draft, Data curation. YL: Data curation, Formal analysis, Writing – original draft. FL: Writing – original draft, Methodology, Data curation. XL: Writing – original draft, Formal analysis, Data curation. MQ: Writing – original draft, Formal analysis, Data curation. YB: Writing – original draft, Formal analysis, Data curation. SQ: Writing – original draft, Formal analysis, Data curation. XH: Writing – original draft, Methodology, Data curation. FJ: Writing – review & editing, Supervision, Project administration, Investigation, Conceptualization. Q-LZ: Investigation, Project administration, Supervision, Writing – review & editing. NG: Writing – review & editing, Supervision, Project administration, Investigation.
