# Supplementary material for: Sex disparities of the effect of the COVID-19 pandemic on mortality among patients living with tuberculosis in the United States
Source: Front Public Health. 2024 Jun 18;12:1413604. doi: 10.3389/fpubh.2024.1413604 (PMC11217309; doi:10.3389/fpubh.2024.1413604)
Supplement: Supplementary file 1 [file Data_Sheet_1.docx]

***Supplementary Material***

**Sex** **disparities of the effect of the COVID-19 pandemic on mortality among patients living with tuberculosis in the United States**

1. **Supplementary Tables and Figures**

## 1.1 Supplementary Tables

**Supplementary Table 1.** Model parameters in mortality among U.S. adults living with TB, 2006-2021.

**Supplementary Table 2.** Annual percentage change (APC) of TB-related mortality in U.S. adults, 2006-2021.

**Supplementary Table 3.** Annual percentage changes (APC) of TB-related mortality in U.S. adults, by age and further stratified by sex, 2006-2021.

**Supplementary Table 4.** Age-standardized mortality rate and annual percentage change (APC) in TB-related mortality among U.S. adults by race/ethnicity, 2006-2020.

## 1.2 Supplementary Figures

**Supplementary Figures 1.** Temporal trends of mortality in general population and excess mortality during the COVID-19 pandemic, by sex, further stratified by Age.

**Supplementary Figures 2.** Observed and predicted age-standardized mortality rates for TB-related deaths before and during the COVID-19 pandemic among different sex and age subgroups.

**Supplementary Figures 3.** Observed and predicted age-standardized mortality rates for TB-related deaths before and during the COVID-19 pandemic by race/ethnicity.

**Supplementary Table 1. Model parameters in TB-related mortality among adults in the U.S., 2006-2021.**

|  | 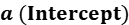  **[95%CI]** | 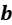  **[95% CI]** | 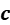 **[95%CI]** | 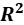**^*^** |
| --- | --- | --- | --- | --- |
| **Overall** | | | | |
| Total**^∭^** | 0.75  [0.71,0.79] | -0.05  [-0.06,-0.04] | 0.002  [0.001,0.003] | 0.96 |
| **Age** | | | | |
| 25-44 years**^∭^** | 0.12  [0.09,0.15] | -0.01  [-0.02,-0.002] | 0.001  [0,0.001] | 0.50 |
| 45-64 years**^∭^** | 0.50  [0.46,0.55] | -0.02  [-0.04,-0.01] | 0.001  [0,0.002] | 0.87 |
| ≥65 years**^∭^** | 2.53  [2.40,2.67] | -0.17  [-0.22,-0.13] | 0.01  [0.004,0.01] | 0.96 |
| **Sex** | | | | |
| Female**^∭^** | 0.49  [0.45,0.53] | -0.03  [-0.04,-0.02] | 0.0001  [0.0002,0.002] | 0.92 |
| Male**^∭^** | 1.09  [1.03,1.16] | -0.07  [-0.09,-0.05] | 0.003  [0.002,0.004] | 0.95 |
| **Race and ethnicity** | | | | |
| Non-Hispanic Whites**^∭^** | 0.44  [0.42,0.46] | -0.03  [-0.03,-0.02] | 0.001  [0.0005,0.001] | 0.97 |
| Non-Hispanic Blacks**^∭^** | 1.82  [1.64,2.00] | -0.17  [-0.23,-0.12] | 0.007  [0.003,0.01] | 0.94 |
| Hispanics**^∭^** | 1.53  [1.35,1.72] | -0.12  [-0.17,-0.06] | 0.004  [0.001,0.01] | 0.88 |
| Non-Hispanic AI/AN**^∭^** | 3.49  [2.44,4.54] | -0.08  [-0.41,0.24] | -0.01  [-0.03,0.02] | 0.68 |
| Non-Hispanic Asians**^∭^** | 3.22  [2.83,3.60] | -0.22  [-0.34,-0.11] | 0.01  [0.001,0.02] | 0.84 |
| **Female by age** | | | | |
| 25-44 years**^∬^** | 0.06  [0.05,0.08] | -0.001  [-0.003,0.001] | -- | 0.10 |
| 45-64 years**^∭^** | 0.21  [0.18,0.25] | 0.002  [-0.01,0.01] | -0.0004  [-0.001,0.0003] | 0.62 |
| ≥65 years**^∭^** | 1.90  [1.76,2.04] | -0.15  [-0.19,-0.10] | 0.006  [0.003,0.009] | 0.95 |
| **Male by age** | | | | |
| 25-44 years**^∭^** | 0.18  [0.14,0.21] | -0.02  [-0.03,-0.008] | 0.001  [0.0003,0.002] | 0.60 |
| 45-64 years**^∭^** | 0.81  [0.74,0.88] | -0.05  [-0.07,-0.03] | 0.002  [0.001,0.003] | 0.89 |
| ≥65 years**^∭^** | 3.53  [3.30,3.75] | -0.23  [-0.30,-0.16] | 0.01  [0.005,0.01] | 0.94 |

^*^
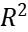
: the goodness of fit, which is used to describe the fitting degree of the regression equation to the observed value. The value range of
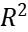
 is [0,1]. The closer
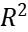
 is to 1, the better the fitting degree is.

^∬^
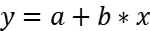
 ; ∭
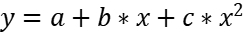


**Supplementary Table 2.** Annual percentage change (APC) of TB-related mortality among U.S. adults, overall, by age and sex, between 2006 and 2021.

|  | **Year** | **Trend Segment**  **APC [95% CI]** |
| --- | --- | --- |
| **Overall** | | |
|  | 2006-2015  2015-2021 | -4.6*[-6.0 to -3.7]  2.2 [-0.1 to 4.5] |
| **Age Group** | | |
| 25-44 years | 2006-2021 | -0.4[-3.1 to 2.4] |
| 45-64 years | 2006-2019  2019-2021 | -2.8* [-3.7 to -1.9]  16.8[-2.2 to 39.5] |
| ≥65 years | 2006-2014  2014-2021 | -5.7* [-6.8 to -4.5]  0.5[-1.0 to 1.9] |
| **Sex** | | |
| Female | 2006-2015  2015-2021 | -5.6* [-7.3 to -3.8]  2.9 [-0.5 to 6.5] |
| Male | 2006-2011  2011-2019  2019-2021 | -6.5*[-9.3 to -3.7]  -1.5[-3.2 to 0.3]  6.7[-6.5 to 21.7] |

*P-value ≤ 0.05

**Supplementary Table 3.** Annual percentage changes (APC) of TB-related mortality among U.S. adults, by age and further stratified by sex, 2006-2021.

|  | **Year** | **Trend Segment**  **APC [95% CI]** |
| --- | --- | --- |
| **Females** | | |
| 25-44 years | 2006-2021 | 0.1 [-2.8to3.2] |
| 45-64 years | 2006-2019  2019-2021 | -2.2* [-3.6 to -0.8]  22.8 [-1.7 to 68.7] |
| ≥65 years | 2006-2014  2014-2021 | -7.2* [-8.6 to -5.8]  0.9* [-1.0 to 2.7] |
| **Males** | | |
| 25-44 years | 2006-2012  2012-2021 | -12.1* [-19.0 to -4.5]  6.5* [1.9 to 11.3] |
| 45-64 years | 2006-2014  2014-2021 | -4.7* [-6.9 to -2.4]  1.1 [-1.7 to 4.1] |
| ≥65 years | 2006-2011  2011-2021 | -6.9* [-9.7 to -3.9]  -1.1 [-2.2 to 0] |

*P-value ≤0.05

**Supplementary Table 4.** Age-standardized mortality rate and annual percentage change (APC) in TB-related mortality among U.S. adults by race/ethnicity, 2006-2020.

| **Age-Standardized Mortality Rate Per 100,000 Persons** | | | | | | **Temporal Trend Differences** | |
| --- | --- | --- | --- | --- | --- | --- | --- |
|  | **Pre-Pandemic**  **Epoch, 2006** | **Pre-Pandemic**  **Epoch, 2019** | **Pandemic Epoch 1**  **2020** | | | **Trend Segments** | **APC [95% CI]** |
| **Race/Ethnicity** | **Observed** | **Observed** | **Observed** | **Predicted  [95% CI]** | **% Difference^†^** |  |  |
| Non-Hispanic Whites | 0.42 | 0.28 | 0.30 | 0.27  [0.25-0.29] | 10.29 | 2006-2016  2016-2020 | -4.3[-5.1 to -3.5]  2.4[-0.9 to 5.8] |
| Non-Hispanic Blacks | 1.68 | 0.60 | 0.82 | 0.78  [0.60-0.96] | 6.06 | 2006-2015  2015-2020 | -8.4*[-11.1 to -5.6]  1.1[-6.0 to 8.7] |
| Hispanics | 1.52 | 0.63 | 0.87 | 0.77  [0.59-0.96] | 12.18 | 2006-2020 | -4.3*[-5.7 to -5.7] |
| Non-Hispanic AI/AN | 3.44 | 1.40 | 1.87 | 1.0  [0.01-2.11] | 76.25 | 2006-2020 | -6.0[-8.4 to -3.5] |
| Non-Hispanic Asians | 3.10 | 1.96 | 1.91 | 1.87  [1.49-2.26] | 1.93 | 2006-2020 | -3.4[-4.7 to -2.2] |

^†^Denotes % difference between predicted and observed values.

*P-value ≤0.05

Non-Hispanic AI/AN, Non-Hispanic American Indian/Alaska Native

**Supplementary Figure 1.** Temporal trends of all-cause mortality in general population and excess mortality during the COVID-19 pandemic, by sex, further stratified by age. (A) Overall population, (B) Female, (C) Male, (D) Aged at 25-44 years old in overall population, (E) Aged at 25-44 years old in female, (F) Aged at 25-44 years old in male, (G) Aged at 45-64 years old in overall population, (H) Aged at 45-64 years old in female, (I) Aged at 45-64 years old in male, (J) Aged 65 years and above in overall population, (K) Aged 65 years and above in female, (L) Aged 65 years and above in male. The mortality rates (per 100,000 persons) in general population increased across all subgroups during the pandemic. The observed mortality rates were above the predicted mortality rates (dashed horizontal line). Depicted by purple bars, the excess mortality associated with COVID-19 was more pronounced in people aged 45 years, especially for people older than 65 years during the pandemic.

**
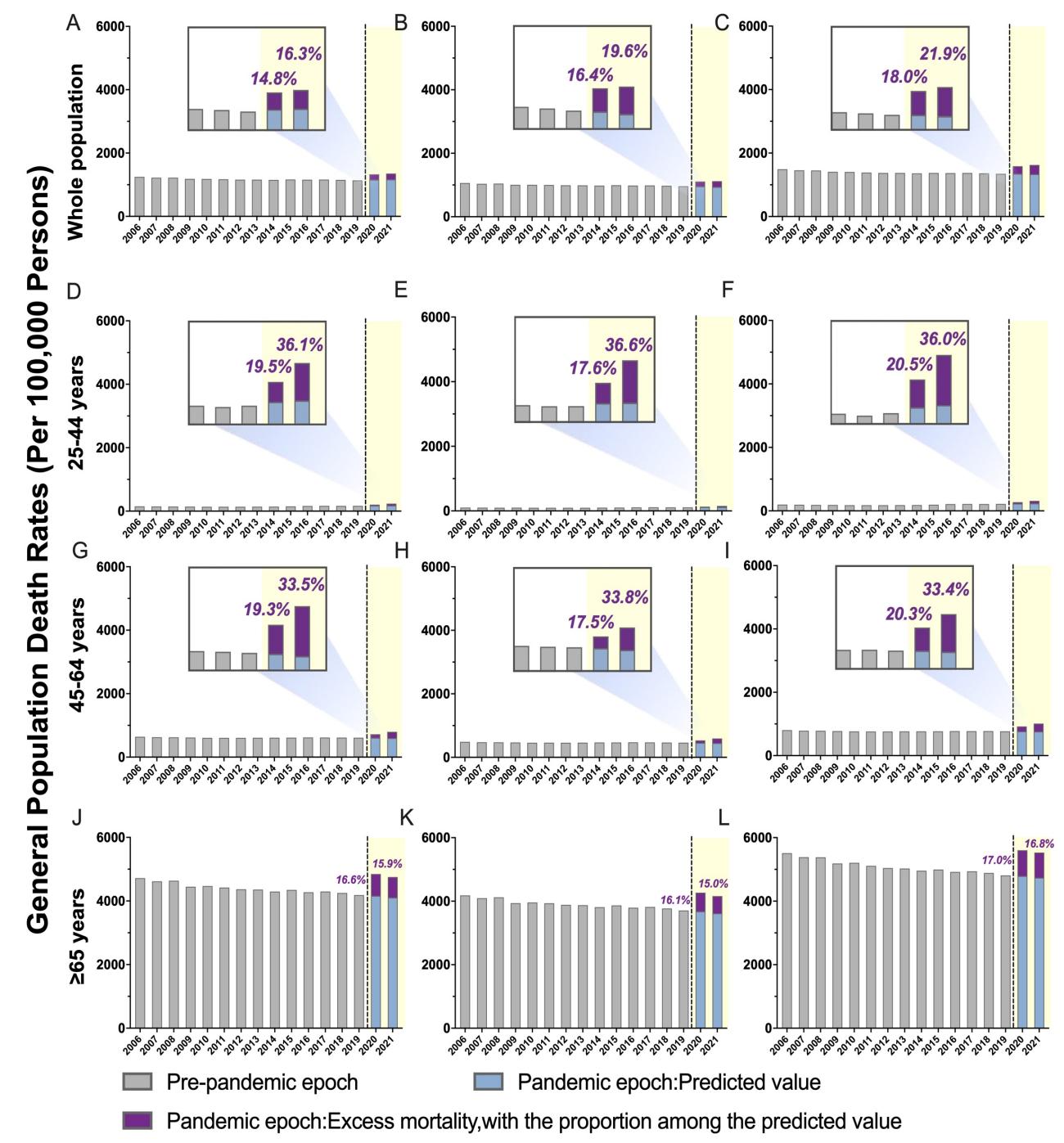
**

**Supplementary Figure 2.** Observed and predicted age-standardized mortality rates for TB-related deaths before and during the COVID-19 pandemic among different sex and age subgroups.

**
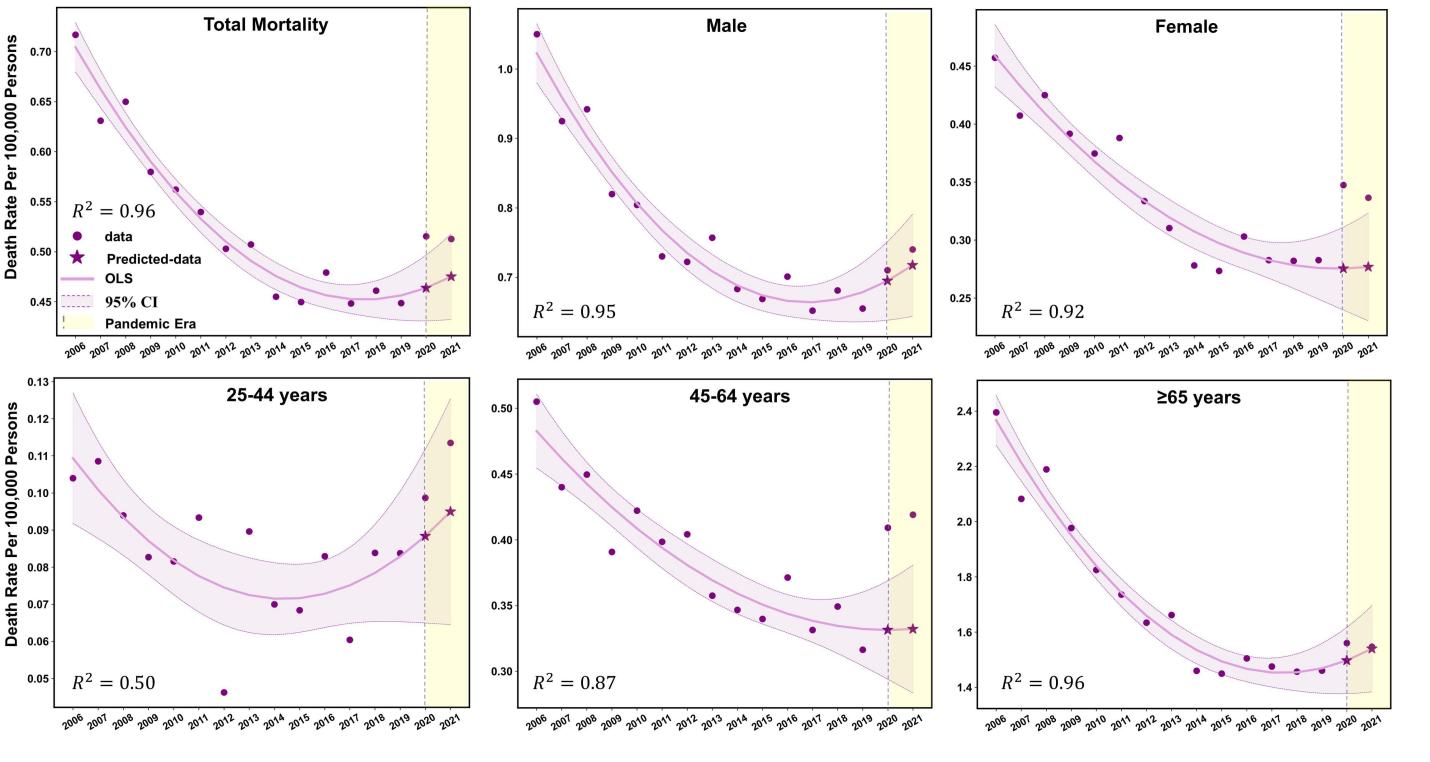
**

**Supplementary Figure 3.** Observed and predicted age-standardized mortality rates for TB-related deaths before and during the COVID-19 pandemic by race/ethnicity.

**
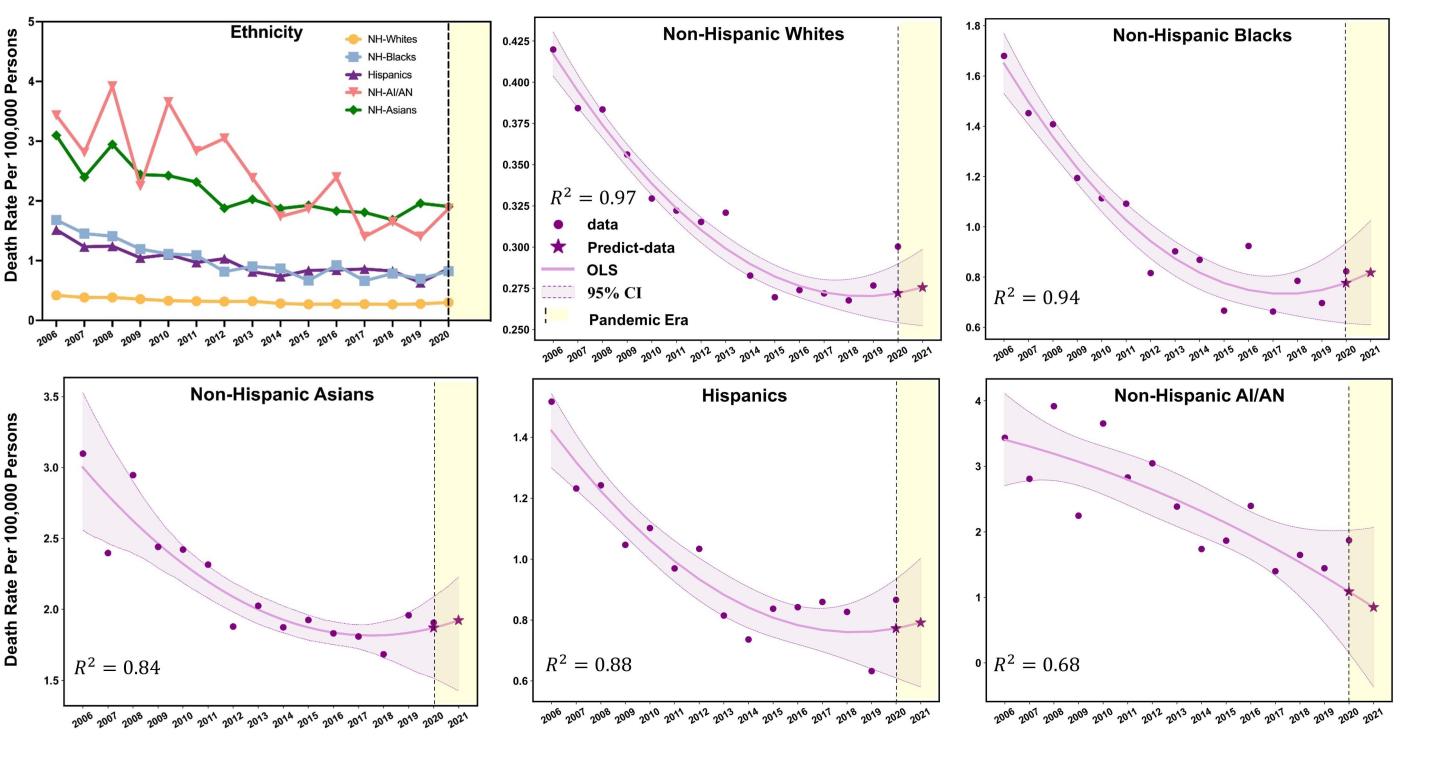
**
